# *Fomitopsis betulina (*formerly *Piptoporus betulinus)*: the Iceman’s polypore fungus with modern biotechnological potential

**DOI:** 10.1007/s11274-017-2247-0

**Published:** 2017-04-04

**Authors:** Małgorzata Pleszczyńska, Marta K. Lemieszek, Marek Siwulski, Adrian Wiater, Wojciech Rzeski, Janusz Szczodrak

**Affiliations:** 1 Department of Industrial Microbiology, Maria Curie-Skłodowska University, Akademicka 19, 20-033 Lublin, Poland; 2 Department of Medical Biology, Institute of Rural Health, Jaczewskiego 2, 20-095 Lublin, Poland; 3 Department of Virology and Immunology, Maria Curie-Skłodowska University, Akademicka 19, 20-033 Lublin, Poland; 4 Department of Vegetable Crops, Poznań University of Life Sciences, Dąbrowskiego 159, 60-594 Poznań, Poland

**Keywords:** Biological activity, Cultivation, *Fomitopsis betulina*, Phytochemistry, *Piptoporus betulinus*

## Abstract

Higher Basidiomycota have been used in natural medicine throughout the world for centuries. One of such fungi is *Fomitopsis betulina* (formerly *Piptoporus betulinus*), which causes brown rot of birch wood. Annual white to brownish fruiting bodies of the species can be found on trees in the northern hemisphere but *F. betulina* can also be cultured as a mycelium and fruiting body. The fungus has a long tradition of being applied in folk medicine as an antimicrobial, anticancer, and anti-inflammatory agent. Probably due to the curative properties, pieces of its fruiting body were carried by Ötzi the Iceman. Modern research confirms the health-promoting benefits of *F. betulina*. Pharmacological studies have provided evidence supporting the antibacterial, anti-parasitic, antiviral, anti-inflammatory, anticancer, neuroprotective, and immunomodulating activities of *F. betulina* preparations. Biologically active compounds such as triterpenoids have been isolated. The mushroom is also a reservoir of valuable enzymes and other substances such as cell wall (1→3)-α-d-glucan which can be used for induction of microbial enzymes degrading cariogenic dental biofilm. In conclusion, *F. betulina* can be considered as a promising source for the development of new products for healthcare and other biotechnological uses.

## Introduction

In 1991, a mummified body was discovered in the Val Senales glacier in Italy. The man (named Ӧtzi the Iceman), who lived 5300 years ago, carried two fragments of a fruiting body of *Fomitopsis betulina* (formerly *Piptoporus betulinus*). Some scientists believe that Ӧtzi might have used the fungus for medical purposes (Capasso 1998) and, although the idea arouses some controversy (Pöder 2005), the long tradition of the use of *F. betulina* in folk medicine is a fact (Reshetnikov et al. 2001; Wasser 2010). Infusion from *F. betulina* fruiting bodies was popular, especially in Russia, Baltic countries, Hungary, Romania for its nutritional and calming properties. Fungal tea was used against various cancer types, as an immunoenhancing, anti-parasitic agent, and a remedy for gastrointestinal disorders (Grienke et al. 2014; Lucas 1960; Peintner and Pöder 2000; Semerdžieva and Veselský 1986; Shamtsyan et al. 2004). Antiseptic and anti-bleeding dressings made from fresh *F. betulina* fruiting body were applied to wounds and the powder obtained from dried ones was used as a painkiller (Grienke et al. 2014; Papp et al. 2015; Rutalek 2002).

In the present paper, we have shown the current knowledge of the fungus *F. betulina*, including its lifestyle, chemical composition, and potential in biotechnology.

## Taxonomy and characteristics

*Piptoporus betulinus* (Bull.) P. Karst. (known as birch polypore, birch bracket, or razor strop) is a common Basidiomycota brown rot macrofungus growing on decaying birch wood. Homobasidiomycetes were divided into eight clades. The family Polyporaceae with the genus *Piptoporus* was classified to the polyporoid clade, and then the antrodia clade—the *Fomitopsis-Daedalea-Piptoporus* group comprising brown rot fungi was identified within this clade (Hibbett and Donoghue 2001; Hibbett and Thorn 2001). Further studies of the phylogenetic relationships among members of the antrodia clade revealed polyphyly of the *Fomitopsis* genus and suggested that *P. betulinus* was phylogenetically closer to *Fomitopsis* than to *Piptoporus* (Kim et al. 2005; Ortiz-Santana et al. 2013). Recently, *P. betulinus* (Bull.) P. Karst. has been transferred to *Fomitopsis* (Han et al. 2016) and, according to Index Fungorum (2016), is classified in the genus *Fomitopsis*, family Fomitopsidaceae, order Polyporales, class Agaricomycetes, division Basidiomycota, kingdom Fungi, with the current name *Fomitopsis betulina* (Bull.) B.K. Cui, M.L. Han and Y.C. Dai, comb.nov. (MycoBank no.: MB 812646).

**Fig. 1:**
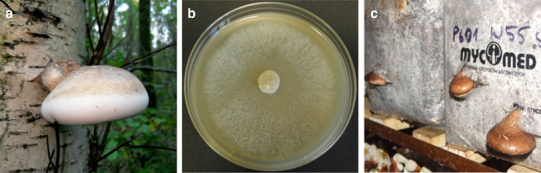
*Fomitopsis betulina*. **a** Basidiocarp of the wild fungus. **b** Mycelium on an agar plate. **c** Mature fruiting body cultured on birch sawdust in artificial conditions. (photographed by M. Siwulski)

*Fomitopsis betulina* is characterized by annual, sessile to effused-reflexed, tough to woody hard basidiocarps, white to tan or pinkish-colored pore surface with mostly small and regular pores. Fruiting bodies grow singly or in small groups, are covered with a laccate, glabrous crust, never zonate, young cream to white, later ochraceous-brown to greyish brown (Fig. 1a). The mycelium of *F. betulina* developing on agar media is white, relatively homogeneous, downy-felt, with regular colony edges (Fig. 1b). The hyphae develop radially. The hyphal system is mostly dimitic. The clamped generative hyphae, 1.5–3.5 µm in diameter, are branched and hyaline whereas the skeletal hyphae with the diameter of 3– 4 µm, are less branched and have thicker walls. No primordia or fruiting bodies of this species were found in vitro (Petre and Tanase 2013). Basidiospores are smooth, hyaline, thin-walled, and cylindrical (Han and Cui 2015; Han et al. 2016; Kim et al. 2005; Schwarze 1993).

The birch polypore grows mainly as a saprophyte on dead trees and occasionally as a parasite of living trees. It occurs in northern temperate forests and parks in Europe, North America, and Asia. The host range of the fungus is restricted exclusively to birch species, e.g. *Betula pendula* Roth., *B. pubescens* Ehrh., *B. papyrifera* Marsh., and *B. obscura* Kotula (Schwarze 1993; Žižka et al. 2010).

## Wood decay

Wood rotting fungi are traditionally divided into white and brown rot species based on the structure and composition of residual wood. Brown rot fungi extensively degrade the carbohydrate fraction of lignocellulose but, in contrast to white rot fungi, leave lignin, although in a modified form. In these fungi, chemical depolymerization of cellulose, which precedes and supports its enzymatic degradation, is very important. They lack ligninolytic peroxidases and usually some other enzymes such as processive cellobiohydrolases used for degradation of crystalline cellulose, but contain H_2_O_2_-generating oxidases and Fe^3+^- and quinone-reducing enzymes used for non-enzymatic depolymerization of polysaccharides (Arantes and Goodell 2014; Baldrian and Valášková 2008; Hori et al. 2013). Modern phylogenetic evidence suggest, however, that there is no sharp distinction between the two groups of fungi (Hori et al. 2013; Riley et al. 2014).

*Fomitopsis betulina* is one of the most common brown rot species but its wood-decaying mechanism has been tested only fragmentarily (Meng et al. 2012) and is still poorly understood. As other fungi of this type, it degrades wood to yield brown, cubical cracks easily broken down. Many factors, including microflora or compounds present in wood, contribute to this complex process (Przybył and Żłobińska-Podejma 2000; Song et al. 2016; Zarzyński 2009). Shang et al. (2013) showed that wood samples decayed by *F. betulina* lost 57% of dry weight (dw) and 74% of holocellulose after 30 days, whereas the fungus growing on wheat straw causes 65% loss of dw within 98 days of culture (Valášková and Baldrian 2006a). A set of enzymes of *F. betulina* involved in the degradation of lignocellulose was characterized in detail by Valášková and Baldrian (2006a, b). The fungus growing on straw produced enzymes with wide substrate specificities: (1→4)-β-endoglucanase, β-glucosidase, (1→4)-β-endoxylanase, (1→4)-β-endomannanase, (1→4)-β-xylosidase, and (1→4)-β-mannosidase. The activities of ligninolytic enzymes and cellobiose dehydrogenase for oxidoreductive cleavage of cellulose were not detected. Similar results were obtained in liquid cultures by Vĕtrovský et al. (2013). When *F. betulina* grew in nature, β-glucosidase and β-mannosidase activity was associated with the fruiting bodies while endopolysaccharidases were detected in colonized wood (Valášková and Baldrian 2006a).

## Cultivation

Carpophores of *F. betulina* from natural habitats or mycelium and culture liquid from submerged cultures were used as raw material to obtain extracts and bioactive substances with medicinal properties (Table 1) (Lomberh et al. 2002). Studies concerning the mycelium growth rate in the presence of various substances (metals, dyes) were conducted mainly on agar media or in liquid cultures (Baldrian and Gabriel 2002; Dresch et al. 2015; Hartikainen et al. 2016). The yield of *F. betulina* mycelium was established in liquid cultures with addition of some agricultural wastes in the studies of Krupodorova and Barshteyn (2015). The enzymatic activity of *F. betulina* was studied in laboratory conditions on agar media (Krupodorova et al. 2014), in liquid cultures (Vĕtrovský et al. 2013), on wheat straw (Valášková and Baldrian 2006a, b), and on *Betula* sp. wood samples (Reh et al. 1986; Shang et al. 2013).

**Table 1:** Biological properties of extracts and compounds isolated from *Fomitopsis betulina*

Biological activity	Mechanism of biological activity	Model [method of study]	Extract^a^	Active compound^a^	References
Bactericidal	Inhibition of bacterial growth	*Bacillus subtilis, Mycobacterium smegmatis, Pseudomonas aeruginosa, Serratia marcescens, Staphylococcus aureus* [zone of inhibition, agar well diffusion assay]	Extracts		Suay et al. (2000)
		*Brucella* sp.[zone of inhibition, agar well diffusion assay]	Benzene extracts	Polyporenic acid (suggested)	Utzig and Fertig (1957)
		*Bacillus* sp., *Rhodococcus equi, S. aureus* [zone of inhibition, disk-diffusion method]	Chloroform extracts		Karaman et al. (2009)
		*B. subtilis, Escherichia coli* [zone of inhibition, agar well diffusion assay]	Dichloromethane extracts		Keller et al. (2002)
		*Bacillus* sp., *R. equi, S. aureus, E. coli* [zone of inhibition, agar well diffusion assay]	Methanol extracts		Karaman et al. (2009), Keller et al. (2002)
		*B. subtilis, Sarcina lutea* [zone of inhibition, agar well diffusion assay]	Ethanol extracts	Polyporenic acid A (suggested)	Kandefer-Szerszeń et al. (1981)
		*B. subtilis, S. lutea, Brucella* sp. [zone of inhibition, agar well diffusion assay]	Ether extracts	Polyporenic acid (suggested)	Kandefer-Szerszeń and Kawecki (1974), Utzig and Fertig (1957)
		*B. subtilis, Enterococcus faecalis, E. coli, S. aureus* [zone of inhibition, agar well diffusion assay, NCCLS-method]		Piptamine isolated from submerged culture of *F. betulina*	Schlegel et al. (2000)
		*B. subtilis, E. coli, S. aureus* [zone of inhibition assay]	Mycelium, culture liquid		Krupodorova et al. (2016)
		*B. subtilis, S. aureus* [zone of inhibition assay]		3β-acetoxy-16α hydroxyl-24-oxo-5α-lanosta-8-ene-21-oic acid	Alresly et al. (2016)
		*E. faecalis* [zone of inhibition assay]	Alkali extract		Vunduk et al. (2015)
Fungicidal	Inhibition of fungal growth	*Saccharomyces cerevisiae, Aspergillus fumigatus*, [zone of inhibition, agar well diffusion assay]	Extracts		Suay et al. (2000)
		*Candida albicans, Kluyveromyces marxianus, Rhodotorula rubra, Sporobolomyces salmonicolor, Penicillium notatum* [zone of inhibition, agar well diffusion assay, NCCLS-method]		Piptamine isolated from submerged culture of *F. betulina*	Schlegel et al. (2000)
Larvicidal	Induction of larva death	*Aedes aegypti* [bioassay]	Dichloromethane extract		Keller et al. (2002)
Antiviral	Protection of CEF cells from *vaccinia virus*	Host/target cells: primary culture of chick embryo fibroblast (CEF) Challenge virus: *vaccinia virus*	Ethanol extracts		Kandefer-Szerszeń et al. (1981)
	Induction of sub stance with properties similar to interferon (hot-stable, stable at pH 2, nondialyzing, insensitive to RNA-se, slightly sensitive to trypsin)	[Plaque formation assays]	Water extracts		Kandefer-Szerszeń and Kawecki (1979)
			Ether extracts	polyporenic acid (suggested)	Kandefer-Szerszeń and Kawecki (1974)
				nucleic acids (RNA and DNA)	Kandefer-Szerszeń et al. (1979)
	Protection of HAT cells from *vaccinia virus* by induction of interferon	Host/target cells: human fibroblast culture (HAT) challenge virus: *vaccinia virus* [plaque formation assays]		RNA	Kawecki et al. (1978)
	Mice protection from lethal infection with TBE	Host/target: Swiss mice Challenge virus: *tick borne encephalitis* (TBE) *virus*	Ethanol extracts		Kandefer-Szerszeń et al. (1981)
	Water extracts induced substance with properties similar to interferon (stable at pH 2, nondialyzing, sensitive to trypsin)	[Neutralization test]	Water extracts		Kandefer-Szerszeń and Kawecki (1979)
			Ether extracts	Polyporenic acid	Kandefer-Szerszeń and Kawecki (1974)
				Nucleic acids (RNA and DNA) (suggested)	Kandefer-Szerszeń et al. (1979), Kawecki et al. (1978)
	Mice protection from lethal infection with HSV-2	host/target: Swiss mice Challenge virus: *herpes simplex virus type 2* (HSV-2) [neutralization test]		RNA	Kawecki et al. (1978)
Anti-inflammatory	Angiotensin I-converting enzyme inhibitory activity		Alkali extract		Vunduk et al. (2015)
	Strong inhibition of 3α-hydroxysteroid dehydrogenase (3α-HSD), hyaluronate lyase and weak inhibition of cyclooxygenase-1 (COX-1)	[Enzyme-based assays: (3α-HSD)-assay according to the method of Penning; N-cetyl-N-trimethylammonium bromide assay according to the method of Ferrante; COX-1 assay]		Polyporenic acid C; (3α,12α,25 S)-12-hydroxy-3-(3-methoxy-1,3- dioxopropoxy)-24-methylene-lanost-8-en-26-oic acid; (3α,12α,25 S)-3-(acetyloxy)-12-hydroxy-24- methylene-lanost-8-en-26-oic acid	Wangun et al. (2004)
	Mice protection from ear edema induction by 12-*O*-tetradecanoylphorbol-13-acetate (TPA)	Mice ear edema model		Polyporenic acid A; polyporenic acid C; (3α,12α,25 S)-3-[(carboxyacetyl)oxy]-12-hydroxy-24-methylene-lanost-8-en-26-oic acid; (3α,12α,25 S)-12-hydroxy-3-[[(3 S)-3-hydroxy-5-methoxy-3-methyl-1,5-dioxopentyl]oxy]-24- methylene-lanost-8-en-26-oic acid; (+)-12α,28-dihydroxy-3α-(30-hydroxy-30-methylglutaryloxy)-24-methyllanosta-8,24(31)-dien-26-oic acid	Kamo et al. (2003)
Antioxidant	Antioxidant capacity	[DPPH scavenging activity, FRAP method]	Water extracts		Vunduk et al. (2015)
	Antioxidant capacity	[DPPH scavenging activity, reducing power, α-carotene bleaching inhibition]		α-, β-, γ-, δ-tocopherols; ascorbic acid; β-carotene; lycopene	Reis et al. (2011)
	Antioxidant capacity	[FRAP method]		p-hydroxybenzoic acid; protocatechuic acid; vanillic acid	Sułkowska-Ziaja et al. (2012)
Immunomodu-lation	Activation of neutrophils to production of reactive oxygen forms	Neutrophils from human peripheral blood [LDCL method]	Water extracts from fruiting bodies and mycelium		Shamtsyan et al. (2004)
Anticancer	Antimigrative properties	Cancer cell lines: A549, HT-29, T47D, TE671 [wound assay]	Ethanol extracts		Pleszczyńska et al. (2016), Zwolińska (2004), Żyła et al. (2005)
		Cancer cell line:TE671 [wound assay]	Ether extracts		Zwolińska (2004)
		Cancer cell lines: A549, C6, HT-29, T47D [wound assay]	Water extracts		Pleszczyńska et al. (2016), Lemieszek et al. (2009)
		Cancer cell lines: A549, HT-29, T47D [wound assay]	Water and ethanol extracts of cultivated fruiting bodies		Pleszczyńska et al. (2016)
	Decrease in tumor cell adhesion	Cancer cell line: LS180 [crystal violet assay]	Ethanol and ether extracts of in vitro grown mycelium		Cyranka et al. (2011)
	Apoptosis induction	Cancer cell line: T47D [ELISA]	Ethanol extracts		Zwolińska (2004)
		Cancer cell line: A549 [ELISA]	Ether extracts		Żyła (2005)
		Cancer cell lines: A549, C6 [ELISA, May Grünwald Giemsa staining]	Water extracts		Lemieszek et al. (2009)
	Cell death induction	Cancer cell lines: A549, T47D, TE671 [May Grünwald Giemsa staining]	Ethanol extracts		Żyła et al. (2005), Zwolińska (2004)
	Decrease in cancer viability	Cancer cell line: HeLa [MTT test]		carboxymethylated (1→3)- -α-D-glucans	Wiater et al. (2011)
	Decrease in cancer viability	Cancer cell line: LS180]MTT test]	Ethanol and ether extracts of in vitro grown mycelium		Cyranka et al. (2011)
	Inhibition of MMP-3, MMP-9, MMP-14	Cancer cell line: A549 [zymography]	Ethanol and ether extracts		Zwolińska (2004)
	Inhibition of MMP-9	Cancer cell line: HT-29 [zymography]	Water extracts		Lemieszek (2008)
	Inhibition of MMP-1, MMP-3, MMP-9	[Hydrolysis of MMP protein substrates—labeled synthetic peptides]		(E)-2-(4-hydroxy-3-methyl-2-butenyl)-hydroquinone	Kawagishi et al. ( 2002)
	Inhibition of MMP-1	[Hydrolysis of MMP protein substrates—labeled synthetic peptides]		polyporenic acid C	Kawagishi et al. (2002)
	Inhibition of cancer cells proliferation	Cancer cell lines: A549, C6, HEp-2, HT-29, Jurkat E6.1, RPMI 8226, T47D, TE671 [MTT test]	Ethanol extracts		Pleszczyńska et al. (2016), Wasyl (2006), Żyła et al. (2005), Zwolińska (2004)
		Cancer cell lines: A549, HT-29, T47D [MTT test]	Ethanol extracts of cultivated fruiting bodies		Pleszczyńska et al. (2016)
		Cancer cell lines: A549, C6, FTC238, HEp-2, HeLa, HT-29, Jurkat E6.1, RPMI 8226, SK-N-AS, T47D, TE671 [MTT test]	Ether extract		Wasyl (2006), Kaczor et al. (2004), Zwolińska (2004)
		Cancer cell lines: A549, C6, HT-29, Jurkat E6.1, T47D [MTT test]	Water extracts		Pleszczyńska et al. (2016), Lemieszek et al. (2009), Zwolińska (2004)
		Cancer cell lines: A549, HT-29, T47D [MTT test]	Water extracts of cultivated fruiting bodies		Pleszczyńska et al. (2016)
		Cancer cell lines: A549, T47D [MTT test]		Polyporenic acid A	Zwolińska (2004)
	Inhibition of DNA synthesis	Cancer cell line: C6 [BrdU test]	Ethanol extracts		Wasyl (2006)
		Cancer cell lines: A549, C6 [BrdU test]	Water extracts		Lemieszek et al. (2009)
	Alterations in cell cycle progression—accumulation of cancer cells in the “S” phase	Cancer cell line: FTC238 [flow cytometry]	Ether extract		Kaczor et al. (2004)
	Inhibition of cancer cell growth	Mouse sarcoma S-37 [not given]	Extracts		Blumenberg and Kessler (1963)
	Tumor size reduction by induction of cancer cell necrolysis and inhibition of metastases	Female dogs with adenocarcinoma mammae, adenocarcinoma solidum, adenocarcinoma papilliferum [histopathological examination after Hansen staining]	Water extracts	Pentacyclic triterpenes (suggested)	Wandokanty et al. (1954; 1955)
	Tumor size reduction and inhibition of bleeding from the genital tract	Female dogs with Sticker’s sarcoma [per vaginal examination]	Ethanol extracts	Pentacyclic triterpenes (suggested)	Utzig and Samborski (1957)
Neuroprotec-tion	Protection of neurons against damage induced by cisplatine, trophic stress, excitotoxicity	Mouse neurons—10-day old [LDH test]	Ethanol and ether extracts		Wasyl (2006)

Cancer cell lines: A549—human Caucasian lung carcinoma, C6—rat glioma, FTC238—human thyroid carcinoma, HeLa—human cervical adenocarcinoma, Hep-2 (HeLa derivative)—human cervix carcinoma, HT-29—human colon adenocarcinoma, Jurkat E6.1—human T-cell leukemia, LS180—human colorectal adenocarcinoma, RPMI 8226—human multiple myeloma, SK-N-AS—human neuroblastoma, T47D—human breast ductal carcinoma, T671—human rhabdomyosarcoma/medulloblastoma

^a^Extracts/compounds were isolated from fruiting bodies of wild growing *F. betulina*, unless otherwise indicated

*3α-HSD* 3-α hydroxysteroid dehydrogenase, *BrdU* − 5-bromo-2’-deoxyuridine, *COX-1* cyclooxygenase-1, *DPPH* 2,2-diphenyl-1-picrylhydrazyl, *ELISA* enzyme-linked immunosorbent assay, *FRAP* ferric ion reducing antioxidant power, *LDCL* luminol-dependent chemiluminescence, *LDH* lactate dehydrogenase, *MTT* methylthiazolyldiphenyl-tetrazolium bromide, *NCCLS* National Committee for Clinical Laboratory Standards

There are limited data on small- or large-scale cultivation of this species in which carpophores could be obtained in controlled conditions. The first such report referring to outdoor log cultivation of *F. betulina* on *Betula davurica* Pallas originated from Korea (Ka et al. 2008). Logs with a diameter of 8–18 cm and length of 107–135 cm were inoculated and then cultured in natural conditions. The yield obtained was in the range from 212 to 1298 g fresh weight (1–2 mushrooms per log). Development of fruiting bodies took an average of 18 months. The ratio of log yield was estimated at 2.8–6.1%. The only report on indoor production of *F. betulina* fruiting bodies was given by Pleszczyńska et al. (2016). In the study, four strains of *F. betulina* isolated from natural habitats were applied. Their mycelia were inoculated into birch sawdust supplemented with organic additives. Mature fruiting bodies weighing from 50 to 120 g were obtained from only one strain, after 3–4 months of the cultivation in artificial conditions (Fig. 1c). The biological efficiency ranged from 12 to 16%. It was shown that extracts isolated from cultivated and naturally grown *F. betulina* fruiting bodies had comparable biological activity (Table 1).

## Biotechnological uses

### Phytochemistry and pharmacological activity

Comprehensive analyses of the chemical composition of the *F. betulina* fruiting body carried out under different conditions (Grishin et al. 2016; Hybelbauerová et al. 2008; Reis et al. 2011) revealed the presence of 17 fatty acids, in it 22% saturated and 78% unsaturated (mainly oleic and linoleic acid); sugars (d-arabinitol, d-mannitol and α,α trehalose); biomolecules with antioxidant properties (tocopherols—0.578 mg/100 g dw, mainly β and γ; ascorbic acid—87.5 mg/100 g dw; β-carotene and lycopene). Among other identified compounds were betulinic acid, betulin, lupeol, fomefficinic acid, ergosterol peroxide, and 9,11-dehydroergosterol peroxide (Alresly et al. 2016; Jasicka-Misiak et al. 2010). Total content of phenolics was determined on 14 or 35 mg GAE/g dw whereas phenolic acids were not detected (Reis et al. 2011; Sułkowska-Ziaja et al. 2012). Product of hydrodistillation of *F. betulina* fruiting bodies contained numerous volatile mono- and sesquiterpenes. Several compounds found, (+)-α-barbatene, (−)-β-barbatene, daucene and isobazzanene, have not been previously reported from other mushrooms. Alcohols, 3-octanol and 1-octen-3-ol, were the main flavour constituents of the fungus (Rapior et al. 1996; Rösecke et al. 2000).

Although some authors considered young specimens of *F. betulina* edible (Wasson 1969), the fungus value is not the result of nutritional but therapeutic properties. The overview of the available literature concerning medical potential of birch polypore was presented in Table 1. Referring to the folk uses of the birch polypore, most of the presented research was based on crude extracts, which often have greater bioactivity than isolated constituents at an equivalent dose. This phenomenon is explained by mostly synergistic interactions between compounds present in mixtures. Furthermore, extracts often contain substances that inhibit multi-drug resistance and therefore further increase the effectiveness of the active substances. Particularly noteworthy among the wide variety of biological activities of *F. betulina* extract, are properties proved in in vivo studies, e.g. the efficacy of water and ethanol extracts in treatment of the genital tract in dogs (Utzig and Samborski 1957; Wandokanty et al. 1954, 1955) or mice protection from lethal infection with the TBE virus by water, ethanol, and ether extracts (Kandefer-Szerszeń et al. 1981; Kandefer-Szerszeń and Kawecki 1974, 1979). The broad spectrum of antiviral and antimicrobial activity of *F. betulina* extracts proved by a number of research teams in different models based on different techniques deserves special attention as well (see references cited in Table 1). Recently, Stamets (2011, 2014) has invented formulations prepared from different medicinal mushrooms including *F. betulina*, which are useful in preventing and treating viral and bacterial diseases, i.e. herpes, influenza, SARS, hepatitis, tuberculosis, and infections with *E. coli* and *S. aureus* .

**Fig. 2:**
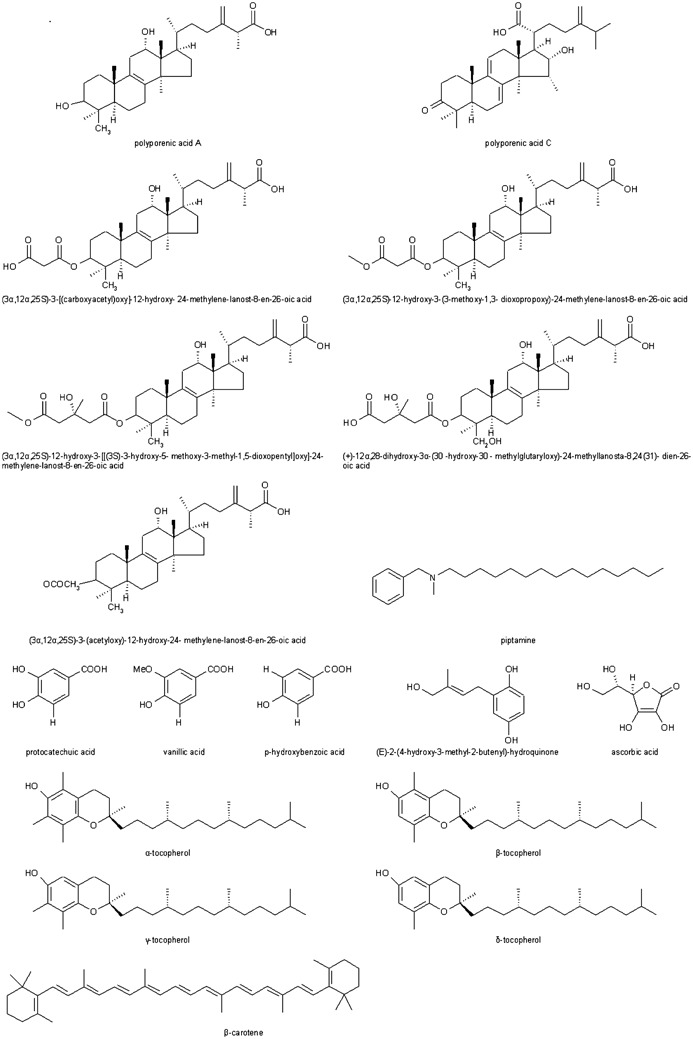
Chemical structures of bioactive compounds isolated from *F. betulina*

Some pure compounds corresponding to the bioactivity of the birch polypore were also identified (Fig. 2). They belong to several chemical classes but the greatest attention was paid to small molecular weight secondary metabolites, especially triterpenoids. Kamo et al. (2003) isolated several triterpenoid carboxylic acids with a lanostane skeleton, e.g. polyporenic acids and their derivatives (Table 1). In in vivo tests, the substances suppressed TPA-induced mouse ear inflammation up to 49–86% at the dose of 0.4 µM/ear. Alresly et al. (2016) purified one previously unknown (identified as 3β-acetoxy-16α hydroxyl-24-oxo-5α-lanosta-8-ene-21-oic acid) and ten known triterpenes from ethyl acetate extract of fruiting bodies of the fungus. The new compound showed anti-gram-positive bacteria activity. The medicinal activity of some triterpenoids tested was examined more accurately. It was shown that polyporenic acid C, just like another compound isolated from *F. betulina*, i.e. (E)-2-(4-hydroxy-3methyl-2-butenyl)-hydroquinone, had inhibitory activity against some matrix metalloproteinases (MMP), with IC_50_ values (concentration causing inhibition by 50% compared to control) in the range from 23 to 128 µM (Kawagishi et al. 2002). Polyporenic acid C and three other *F. betulina* triterpenoids (Table 1) showed anti-inflammatory and antibacterial activity by strong inhibition of 3α-hydroxysteroid dehydrogenase and bacterial hyaluronate lyase activity, respectively (Wangun et al. 2004).

In their search for fungal antimicrobial substances, Schlegel et al. (2000) isolated another valuable compound—piptamine, N-benzyl-N-methylpentadecan-1-amine from submerged culture of *F. betulina* Lu 9-1. It showed activity against gram-positive bacteria (MIC, minimum inhibitory concentration, values in the range from 0.78 to 12.5 µg/ml) and yeasts including *Candida albicans* (MIC 6.25 µg/ml).

Polysaccharides from higher basidiomycota mushrooms have been usually considered to be the major contributors of their bioactivity. However, birch polypore polysaccharides have not yet been sufficiently explored, in terms of either the structure or pharmacological activity. It is known that the *Fomitopsis* cell wall contains (1→3)-β-d-glucans in an amount of ca. 52% dw (Jelsma and Kreger 1978; Grün 2003). They are built from β-d-glucopyranose units connected with (1→3)-linkages in the main chain, with (1→3)-β-d linked side branches. However, there are no reports about their biological activities. Another polysaccharide isolated from the birch polypore was water-insoluble, alkali-soluble (1→3)-α-d-glucan. Although α-glucans are believed to be biologically inactive, its carboxymethylated derivative showed moderate cytotoxic effects in vitro (Wiater et al. 2011).

## Miscellaneous applications

With the knowledge of the mechanisms of action of brown rot decay, there are possibilities of new applications of these fungi in biotechnology. The enzymatic and non-enzymatic apparatus for lignocellulose degradation can be used for bioprocessing of biomass towards fuels and chemicals (Arantes et al. 2012; Giles and Parrow 2011; Ray et al. 2010). Brown rot fungi, including *F. betulina*, were tested for bioleaching of heavy metals (Cu, Cr, and As) from wood preservatives due to accumulation of metal-complexing oxalic acid (Sierra Alvarez 2007). Production of biomass degrading enzymes, for instance cellulases, hemicellulases, amylases, etc., was also studied (Krupodorova et al. 2014; Valášková and Baldrian 2006a, b).

The cell wall of *F. betulina* can be a source of useful polysaccharides, e.g. water-insoluble, alkali-soluble α-glucans (Grün 2003; Jelsma and Kreger 1979). (1→3)-α-d-glucans whose main chain contains 84.6% of (1→3)-linked α-d-glucopyranose in addition to 6% of (1→4)-linked units were purified and characterized by Wiater et al. (2011). Another polysaccharide, named piptoporane I, was extracted and purified by Olennikov et al. (2012). This α-glucan was built from residues of (1→3)-α-d-glucopyranose with occasional branching by single residues of β-d-glucopyranose at the C_6_ position (17.3%). It has been shown that fungal (1→3)-α-d-glucans, including that from *F. betulina*, effectively induce the production of microbial (1→3)-α-glucanases (mutanases), i.e. enzymes that have potential in dental caries prevention. (1→3),(1→6)-α-d-Glucans (mutans) synthesized by mutans streptococci are key structural and functional constituents of dental plaque matrix; therefore, they seem to be a good target for enzymatic anti-caries strategy (Pleszczyńska et al. 2015). However, streptococcal glucans are difficult to use as inducers of mutanases because of the low yield and structural variation. Birch polypore α-glucan, whose amount in the cell wall of *F. betulina* reaches even 44–53% dw (Grün 2003), can be used to replace streptococcal glucans (Wiater et al. 2008).

## Conclusions and outlook

The *F. betulina* fungus has been widely used and appreciated in folk medicine, and modern pharmacological studies have confirmed its potential indicating significant antimicrobial, anticancer, anti-inflammatory, and neuroprotective activities. The possibility of successful cultivation thereof in artificial conditions additionally promotes the applicability of the fungus. However, compared with other polypore fungi, the research on *F. betulina* is less developed; for instance, little is known about its lifestyle, including the wood degradation strategy. Moreover, most of the bioactivity studies have been performed using crude extracts; hence, only a few of the effects have been associated with the active substances identified, e.g. antibacterial activities with piptamine or polyporenic acids. With a few exceptions, we still do not know the mechanisms underlying the biological activities. Verification of biological activities in in vivo and clinical studies is also required. The further research could contribute to better exploitation of the *F. betulina* application potential.

## References

[CR1] Alresly Z, Lindequist U, Lalk M, Porzel A, Arnold N, Wessjohann LA (2016). Bioactive triterpens from the fungus *Piptoporus betulinus*. Rec Nat Prod.

[CR2] Arantes V, Goodell B (2014). Current understanding of brown-rot fungal biodegradation mechanisms: a review. In: deterioration and protection of sustainable biomaterials. ACS Symp Ser.

[CR3] Arantes V, Jellison J, Goodell B (2012). Peculiarities of brown-rot fungi and biochemical Fenton reaction with regard to their potential as a model for bioprocessing biomass. Appl Microbiol Biotechnol.

[CR4] Baldrian P, Gabriel J (2002). Intraspecific variability in growth response to cadmium of the wood-rotting fungus *Piptoporus betulinus*. Mycologia.

[CR5] Baldrian P, Valášková V (2008). Degradation of cellulose by basidiomycetous fungi. FEMS Microbiol Rev.

[CR6] Blumenberg F, Kessler F (1963). Inhibition of the growth of mouse sarcoma S-37 by the birch fungus (*Polyporus betulinus*). Arzneimittelforschung.

[CR7] Capasso L (1998). 5300 years ago, the Ice man used natural laxatives and antibiotics. Lancet.

[CR8] Cyranka M, Grąz M, Kaczor J (2011). Investigation of antiproliferative effect of ether and ethanol extracts of Birch polypore medicinal mushroom, *Piptoporus betulinus* (Bull.: Fr.) P. Karst. (Higher Basidiomycetes) in vitro grown mycelium. Int J Med Mushrooms.

[CR9] Dresch P, D’Aguanno MN, Rosam K, Grienke U, Rollinger JM, Peintner U (2015). Fungal strain matters: colony growth and bioactivity of the European medicinal polypores *Fomes fomentarius, Fomitopsis pinicola* and *Piptoporus betulinus*. AMB Express.

[CR10] Fungorum I (2016) http://www.indexfungorum.org. Accessed 29 Nov 2016

[CR11] Giles R, Parrow M (2011) Lignocellulosic treatments and applications thereof. United States Patent Application Publication US 20110008384 A1

[CR12] Grienke U, Zöll M, Peintner U, Rollinger JM (2014). European medicinal polypores—A modern view on traditional uses. J Ethnopharmacol.

[CR13] Grishin AA, Lutskii VI, Penzina TA, Dudareva LV, Zorina NV, Polyakova MS, Osipenko SN (2016). Composition of the supercritical CO_2_ extract of the fungus *Piptoporus betulinus*. Chem Nat Compd.

[CR14] Grün CH (2003) Structure and biosynthesis of fungal α-glucans. Dissertation, University of Utrecht

[CR15] Han ML, Cui BK (2015). Morphological characters and molecular data reveal a new species of *Fomitopsis* (*Polyporales*) from southern China. Mycoscience.

[CR16] Han ML, Chen YY, Shen LL, Song J, Vlasak J, Dai YC, Cui BK (2016). Taxonomy and phylogeny of the Brown-rot Fungi: *Fomitopsis* and its related genera. Fungal Divers.

[CR17] Hartikainen ES, Miettinen O, Hatakka A, Kahkonen MA (2016). Decolorization of six synthetic dyes by fungi. Am J Environ Sci.

[CR18] Hibbett DS, Donoghue MJ (2001). Analysis of character correlations among wood decay mechanisms, mating systems, and substrate ranges in homobasidiomycetes. Syst Biol.

[CR19] Hibbett DS, Thorn RG, McLaughlin DJ, McLaughlin EG, Lemke PA (2001). Basidiomycota: homobasidiomycetes. Systematics and evolution, the Mycota VII Part B.

[CR20] Hori C, Gaskell J, Igarashi K, Samejima M, Hibbett D, Henrissat B, Cullen D (2013). Genomewide analysis of polysaccharides degrading enzymes in 11 white-and brown-rot Polyporales provides insight into mechanisms of wood decay. Mycologia.

[CR21] Hybelbauerová S, Sejbal J, Dračίnskỳ M, Hahnová A, Koutek B (2008). Chemical constituents of *Stereum subtomentosum* and two other bitch-associated basidiomycetes: an interspecies comparative study. Chem Biodivers.

[CR22] Jasicka-Misiak I, Lipok J, Swider IA, Kafarski P (2010). Possible fungistatic implications of betulin presence in betulaceae plants and their hymenochaetaceae parasitic fungi. Z Naturforschung C.

[CR23] Jelsma J, Kreger DR (1978). Observations of the cell-wall compositions of the bracket fungi *Laetiporus sulphureus* and *Piptoporus betulinus*. Arch Microbiol.

[CR24] Jelsma J, Kreger DR (1979). Polymorphism in crystalline (1→3)-α-d-glucan from fungal cell-walls. Carbohydr Res.

[CR25] Ka K-H, Ryu S-R, Lee B-H, Yoon K-H, Bak W-C (2008). Log cultivation of the birch fungus *Piptoporus betulinus* using *Betula davurica*. . Korean J Mycol.

[CR26] Kaczor J, Klecha IM, Rzeski W, Paduch R, Zdzisińska B, Pożarowski P, Kandefer-Szerszeń M (2004). Extract from *Piptoporus betulinus* Bull. Fr. suppresses human tumor cell growth. Post Fitoter.

[CR27] Kamo T, Asanoma M, Shibata H, Hirota M (2003). Anti-inflammatory lanostane-type triterpene acids from *Piptoporus betulinus*. J Nat Prod.

[CR28] Kandefer-Szerszeń M, Kawecki Z (1974). Ether extracts from the fruiting body of *Piptoporus betulinus* as interference inducers. Acta Microbiol Pol Series A: Microbiologia Generalis.

[CR29] Kandefer-Szerszeń M, Kawecki Z (1979). Water extracts of fungi as source of antiviral substances. Ann UMCS XXXIV.

[CR30] Kandefer-Szerszeń M, Kawecki Z, Guz M (1979). Fungal nucleic acids as interferon inducers. Acta Microbiol Pol Series A: Microbiologia Generalis.

[CR31] Kandefer-Szerszeń M, Kaczor J, Kawecki Z (1981). Fungal extracts as source of antiviral substances. II. Application of the chromatography methods for the isolation of antiviral substances from *Piptoporus betulinus* (Bull. Ex Fr.). Ann UMCS XXXVI.

[CR32] Karaman M, Mimica-Dukic N, Knezevic P, Svircev Z, Matavuly M (2009). Antibacterial properties of selected lignicolous mushrooms and fungi from Northern Serbia. Int J Med Mushrooms.

[CR33] Kawagishi H, Hamajima K, Inoue Y (2002). Novel hydroquinone as a matrix metallo-proteinase inhibitor from the mushroom *Piptoporus betulinus*. Biosci Biotechnol Biochem.

[CR34] Kawecki Z, Kaczor J, Karpińska T, Sujak I, Kandefer-Szerszeń M (1978). Studies of RNA isolated from *Piptoporus betulinus* as interferon inducer. Arch Immunol Ther Exp.

[CR35] Keller C, Maillard M, Keller J, Hostettmann K (2002). Screening of European fungi for antibacterial, antifungal, larvicidal, molluscicidal, antioxidant and free-radical scavenging activities and subsequent isolation of bioactive compounds. Pharm Biol.

[CR36] Kim KM, Yoon Y-G, Jung HS (2005). Evaluation of the monophyly of *Fomitopsis* using parsimony and MCMC methods. Mycology.

[CR37] Krupodorova TA, Barshteyn VY (2015). Alternative substrates for higher mushrooms mycelia cultivation. J Biosci Biotechnol.

[CR38] Krupodorova TA, Ivanova T, Barshteyn VY (2014). Screening of extracellular enzymatic activity of macrofungi. J Microbiol Biotechnol Food Sci.

[CR39] Krupodorova TA, Barshteyn VY, Zabeida EF, Pokas EV (2016). Antibacterial activity of macromycetes mycelia and culture liquid. Microbiol. Biotechnol Lett.

[CR40] Lemieszek MK (2008) The estimation of biological activity of water extracts from *Piptoporus betulinus* and *Inonotus obliquus*. Dissertation, Maria Curie-Skłodowska University, Lublin, Poland

[CR41] Lemieszek MK, Langner E, Kaczor J (2009). *Piptoporus betulinus* (Bull.: Fr.) P. Karst. (*Aphyllophoromycetideae*): in vitro studies. Int J Med Mushrooms.

[CR42] Lomberh ML, Solomko EF, Buchalo AS, Kirchhoff B (2002) Studies of medicinal mushrooms in submerged cultures. In: Sanchez et al. (eds) Mushroom biology and mushroom products. The 4th international conference on mushroom breeding and mushroom products. pp 367–377

[CR43] Lucas EH (1960) Folklore and plant drugs. Papers of the Michigan Academy of Science, Arts, and Letters XLV, 127–136

[CR44] Meng F, Liu X, Wang Q (2012). Identification of wood decay related genes from *Piptoporus betulinus* (Bull. Fr.) Karsten using differential display reverse transcription PCR (DDRT-PCR). Biotechnol Biotechnol Equip.

[CR45] Olennikov DN, Agafonova SV, Rokhin AV, Penzina TA, Borovskii GB (2012). Branched glucan from the fruiting bodies of *Piptoporus betulinus* (Bull.: Fr) Karst. Appl Biochem Microbiol.

[CR46] Ortiz-Santana B, Lindner DL, Miettinen O, Justo A, Hibbett DS (2013). A phylogenetic overview of the antrodia clade (Basidiomycota, Polyporales). Mycologia.

[CR47] Papp N, Rudolf K, Bencsik T, Czégényi D (2015). Ethnomycological use of *Fomes fomentarius* (L.) Fr. and *Piptoporus betulinus* (Bull.) P. Karst. in Transylvania, Romania. Genet Resour Crop Evol.

[CR48] Peintner U, Pöder R, Bortenschlager S, Oeggl K (2000). Ethnomycological remarks on the Iceman’s fungi. The Iceman and his natural environment.

[CR49] Petre CV, Tanase C (2013). Description of the culture characteristics of some lignicolous basidiomycetes species grown on three synthetic media. J Plant Dev.

[CR50] Pleszczyńska M, Wiater A, Janczarek M, Szczodrak J (2015). (1→3)-α-d-Glucan hydrolases in dental biofilm prevention and control: a review. Int J Biol Macromol.

[CR51] Pleszczyńska M, Wiater A, Siwulski M, Lemieszek MK, Kunaszewska J, Kaczor J, Rzeski W, Janusz G, Szczodrak J (2016). Cultivation and utility of *Piptoporus betulinus* fruiting bodies as a source of anticancer agents. World J Microbiol Biotechnol.

[CR52] Pöder R (2005). The Iceman’s fungi: facts and mysteries. Int J Med Mushrooms.

[CR53] Przybył K, Żłobińska-Podejma M (2000). Effects of some bacteria *Pseudomonas* spp. and *Erwinia herbicola* on in vitro growth of *Piptoporus betulinus*. For Pathol.

[CR54] Rapior S, Cavalié S, Andary C, Pélissier Y, Marion C, Bessiére JM (1996). Investigation of some volatile components of seven fresh wild mushrooms (Basidiomycetes). J Essent Oil Res.

[CR55] Ray MJ, Leak DJ, Spanu PD, Murphy RJ (2010). Brown rot fungal early stage decay mechanism as a biological pretreatment for softwood biomass in biofuel production. Biomass Bioenergy.

[CR56] Reh U, Kraepelin G, Lamprecht I (1986). Use of differential scanning calorimetry of structural analysis of fungally degraded wood. Appl Environ Microbiol.

[CR57] Reis FS, Pereira E, Barros L, Sousa MJ, Martins A, Ferreira IC (2011). Biomolecule profiles in inedible wild mushrooms with antioxidant value. Molecules.

[CR58] Reshetnikov SV, Wasser SP, Tan KK (2001). Higher basidiomycota as source of antitumor and immunostimulating polysaccharides. Int J Med Mushrooms.

[CR59] Riley R, Salamov AA, Brown DW (2014). Extensive sampling of basidiomycete genomes demonstrates inadequacy of the white-rot/brown-rot paradigm for wood decay fungi. Proc Natl Acad Sci.

[CR60] Rösecke J, Pietsch M, König WA (2000). Volatile constituents of wood-rotting basidiomycetes. Phytochemistry.

[CR61] Rutalek R (2002). Ethnomykologie–Eine Übersicht. Österr Z Pilzkd.

[CR62] Schlegel B, Luhmann U, Hartl A, Grafe U (2000). Piptamine, a new antibiotic produced by *Piptoporus betulinus* Lu 9–1. J Antibiot.

[CR63] Schwarze FWMR (1993). *Piptoporus betulinus* (Bull.: Fr.) Karsten. Mycologist.

[CR64] Semerdžieva M, Veselský J (1986). Léčivé houby dřive a nyni.

[CR65] Shamtsyan M, Konusova V, Maksimova Y, Goloshchev A, Panchenko A, Simbirtsev A, Petrishchev N, Denisova N (2004). Immunostimulating and anti-tumor action of extracts of several mushrooms. J Biotechnol.

[CR66] Shang J, Yan S, Wang Q (2013). Degradation mechanism and chemical component changes in *Betula platyphylla* wood by wood-rot fungi. BioResources.

[CR67] Sierra Alvarez R (2007). Fungal bioleaching of metals in preservative-treated wood. Process Biochem.

[CR68] Song Z, Kennedy PG, Liew FJ, Schilling S (2016). Fungal endophytes as priority colonizers initiating wood decomposition. Funct Ecol.

[CR69] Stamets PE (2011) Antiviral activity from medicinal mushrooms. United States Patent Application Publication US 20110008384 A1

[CR70] Stamets PE (2014) Antiviral and antibacterial activity from medicinal mushrooms. United States Patent Application Publication US 201440105928 A2

[CR71] Suay I, Arenal F, Asensio FJ (2000). Screening of basidiomycetes for antimicrobial activities. Antonie Van Leeuwenhoek.

[CR72] Sułkowska-Ziaja K, Muszyńska B, Motyl P, Pasko P, Ekiert H (2012). Phenolic compounds and antioxidant activity in some species of polyporoid mushrooms from Poland. Int J Med Mushrooms.

[CR73] Utzig J, Fertig S (1957). Influence of polyporenic acids on the growth of the bacterium of *Brucella*. Med Weter.

[CR74] Utzig J, Samborski Z (1957). Effect of triterpenes present in *Polyporus betulinus* on Sticker’s tumors. Med Weter.

[CR75] Valášková V, Baldrian P (2006). Degradation of cellulose and hemicelluloses by the brown rot fungus *Piptoporus betulinus* – production of extracellular enzymes and characterization of the major cellulases. Microbiology.

[CR76] Valášková V, Baldrian P (2006). Estimation of bound and free fractions of lignocellulose-degrading enzymes of wood-rotting fungi *Pleurotus ostreatus, Trametes versicolor* and *Piptoporus betulinus*. Res Microbiol.

[CR77] Vĕtrovský T, Baldrian P, Gabriel J (2013). Extracellular enzymes of the white-rot fungus *Fomes fomentarius* and purification of 1,4-α-glucosidase. Appl Biochem Biotechnol.

[CR78] Vunduk J, Klaus A, Kozarski M, Petrovic P, Zizak Z, Niksic M, Van Griensven LJLD (2015). Did the Iceman know better? screening of the medicinal properties of the birch polypore medicinal mushroom, *Piptoporus betulinus* (Higher Basidiomycetes). Int J Med Mushrooms.

[CR79] Wandokanty F, Utzig J, Kotz J (1954). The action of hydrolysates of *Poria obliqua* and *Polyporus betulinus* on malignant neoplastic cells. Med Weter.

[CR80] Wandokanty F, Utzig J, Kotz J (1955). The effect of *Poria obliqua* and *Polyporus betulinus* on spontaneous cancer of the dog with respect to breast cancer in dogs. Med Weter.

[CR81] Wangun HVK, Berg A, Hertel W, Nkengfack AE, Hertweck C (2004). Anti-inflammatory and anti-hyaluronate lyase activities of lanostanoids from *Piptoporus betulinus*. J Antibiot.

[CR82] Wasser SP (2010). Medicinal mushroom science: history, current status, future trends, and unsolved problems. Int J Med Mushrooms.

[CR83] Wasson RG (1969). Soma: divine mushroom of immortality.

[CR84] Wasyl A (2006) Evaluation of neurotrophic properties of ethanol and ether extracts from polyporous bracket fungi *Piptoporus betulinus* (Bull. Ex Fr.) P. Karst in in vitro model. Dissertation, Maria Curie-Skłodowska University, Lublin, Poland

[CR85] Wiater A, Szczodrak J, Pleszczyńska M (2008). Mutanase induction in *Trichoderma harzianum* by cell wall of *Laetiporus sulphureus* and its application for mutan removal from oral biofilms. J Microbiol Biotechnol.

[CR86] Wiater A, Paduch R, Pleszczyńska M (2011). α-(1→3)-d-Glucans from fruiting bodies of selected macromycetes fungi and the biological activity of their carboxymethylated products. Biotechnol Lett.

[CR87] Zarzyński P (2009). Correlation between phenolic compounds in wood and its decay by chosen species of saprotrophic and parasitic fungi. For Res Pap.

[CR88] Žižka Z, Vetrovsky T, Gabriel J (2010). Enhancement of autofluorescence of the brown-rot fungus *Piptoporus betulinus* by metal ions. Folia Microbiol.

[CR89] Zwolińska K (2004) Evaluation of anticancer activity of extracts from birch polypore *Piptoporus betulinus* (Bull. ex Fr.) P. Karst. Dissertation, Maria Curie-Skłodowska University, Lublin, Poland

[CR90] Żyła M (2005) Comparison of antitumor activity of extracts from *Piptoporus betulinus, Fomes fomentarius* and *Inonotus obliquus*. Dissertation, Maria Curie-Skłodowska University, Lublin, Poland

[CR91] Żyła M, Rzeski W, Kaczor J, Kandefer-Szerszeń M (2005). Birch tree fungi–a source of substances with therapeutic properties (part II). Med Ogólna.

